# Surgical treatment of esophageal carcinoma with curative intent: analysis of a single center experience

**DOI:** 10.1186/1749-8090-8-52

**Published:** 2013-03-19

**Authors:** Tiziano De Giacomo, Paolo Trentino, Federico Venuta, Socratis Tsagkaropoulos, Pasquale Bartolomeo Berloco, Daniele Diso, Federico Francioni

**Affiliations:** 1 Department of Surgery and Transplantation “P. Stefanini”, University of Rome “Sapienza” Policlinico Umberto I, Rome, Italy; 2 Department of Surgery and Transplantation “P.Stefanini”, University of Rome “Sapienza” Thoracic Surgery, Policlinico Umberto I, Viale Policlinico, Rome, 00164, Italy

## Abstract

**Background:**

We retrospectively reviewed our series of 76 patients who underwent esophagectomy, with curative intent, for esophageal carcinoma over the last 10 years.

**Method:**

The mean age was 60 years ranging between 46 to 76 years. Fifty-seven patients had a squamous cell carcinoma and 19 patients had an adenocarcinoma. In 15 cases induction therapy was accomplished prior to surgery. A narrow gastric tube was used to restore continuity in 74 patients (97.3%). Medical records were reviewed and data analysis was performed.

**Results:**

Peri-operative mortality was 2.6%. Overall survival at 1, 3 and 5 years was 85,5%, 67,7% and 52,7%, respectively, with no significant difference between the squamous cell disease group and the adenocarcinoma group. Although T factor and stage at the time of surgery influenced overall survival, the presence of nodal metastasis had the major impact on survival as confirmed by univariate and multivariate analysis with a 5 year survival rate of 32% regardless of the use or not of adjuvant chemo-radiotherapy and the pathologic stage.

**Conclusions:**

Esophagectomy still represents a valid treatment for esophageal carcinoma in well selected patients. Both pT stage and N stage appear to be the most important factors determining survival for patients with completely resected esophageal carcinoma.

## Background

Cancer of the esophagus, despite the advances in its diagnosis and treatment, remains a highly lethal malignancy that increased dramatically in incidence during the last 3 decades [1]. Surgery is an effective modality treatment, either alone or as a part of a multimodality approach, to obtain a local tumor control and the potential for a long term survival. Reduction of complication and mortality rate after esophagectomy are firmly connected to an appropriate patient selection, meticulous execution of a well-chosen surgical technique, and esophageal surgical experience, which bears a direct relationship to the outcome. However, there are still several controversies in the optimal management of patients with resectable esophageal carcinoma in surgical approach, extent of resection and lymphadenectomy, type of reconstruction and site of the anastomosis. The objective of this study is to review and report our experience performing curative esophagectomy for esophageal carcinoma evaluating the oncologic and functional outcome.

## Methods

We retrospectively reviewed all the esophageal procedures performed with curative intent (n=76) for esophageal carcinoma at the Department of Thoracic Surgery of the University of Rome “Sapienza” between January 1998 and July 2010. There were 50 male and 26 female patients, ranging in age from 46 to 76 years (median age, 60 years). The most common presentation symptom was mild or severe dysphagia.

Pre-operative diagnostic evaluation in all patients was mainly based on endoscopic examination of the esophagus and biopsies were obtained to assess histology. Fifty-seven patients (75%) had a squamous cell carcinoma (SCC) of the mid esophagus and 19 (25%) had an adenocarcinoma (ADC) of the distal esophagus. Whole body computed tomography scan was performed to complete clinical staging, while presence of endobronchial tumor infiltration was excluded in all cases by fiberoptic bronchoscopy. Barium swallow, blood chemistry analysis, liver function and cardio-respiratory evaluation completed the preoperative assessment. Enteral or parenteral nutritional supplementation was provided to patients with poor nutritional status. Bowel preparation was undertaken in all patient prior to surgery. A thoracic epidural catheter was placed for post-operative analgesia.

Platinum based induction therapy regimen was accomplished in 15 patients preoperatively staged as IIB and III disease. The protocol used consisted of three cycles of 5-fluorouracil and cisplatin followed by CT scan re-staging and endoscopy. In 12 patients we observed significant clinical and radiologic down-staging while in the remaining 3 the disease remained stable. All patients underwent surgery 3 to 4 weeks after the end of neo-adjuvant treatment.

All operations were performed with curative intent and accomplished via right fifth intercostal space thoracotomy in the majority of the cases; right video-assisted thoracoscopy was employed in selected case of early-stage esophageal cancer and in patients with high cardio-respiratory risk. Trans-hiatal esophagectomy was also reserved in highly selected patients with stage I or IIA disease. Continuity of alimentary tract was re-established by interposition of a “narrow” gastric tube or a part of colon when the use of stomach was not feasible, prepared and mobilized through a median laparotomy. The conduit was then placed in the posterior mediastinum in the prevertebral space (esophageal bed) and was pulled up to the neck. Left-side cervicotomy and end-to-end complete mechanical anastomosis was always performed using the technique already described in the past [2]. Pyloroplasty was not considered necessary. Two-field radical lymphnode dissection was routinely carried out in all cases.

Data analysis was performed with Statistica 7.0 (StatSoft Inc, Tulsa, OK, USA). Continuous variables are expressed as mean (± standard error) or as median [range]. Student *t*-test and Mann–Whitney *U*-test were used to compare for parametric and non-parametric variables, respectively. Categorical data are expressed as frequencies (percentages) and compared with the chi square test or Fisher exact test. Survival was estimated using the Kaplan-Meier method and comparisons between groups were made with log-rank test. Univariate and multivariate analyses were performed using Cox proportional hazards regression model to look for independent predictors of survival. Statistical significance was assessed at the 5% level.

## Results

Demographics of the patients and histologic diagnosis are presented in Table 1.

**Table 1:** Characteristics and results in patients Cohort (n=76)

	**Overall**	**SCC (n=57)**	**ADC (n=19)**	***P *value**
**Age (years)**	60 [46 – 76]	61 [46–76]	57 [46–71]	0,09
**Sex**				0,78
Male	50 (65,79%)	37 (64,91%)	13 (68,42%)	
Female	26 (34,21%)	20 (35,09%)	6 (31,58%)	
**Esophagectomy**				0,76
Thoracotomy	64 (84,21%)	49 (85,96%)	15 (78,95%)	
VATS	9 (11,84%)	6 (10,53%)	3 (15,79%)	
Transhiatal	3 (3,95%)	2 (3,51%)	1 (5,26%)	
**pT factor**				<0,01
T1	10 (13,16%)	5 (8,77%)	5 (26,32%)	
T2	33 (43.42%)	21 (36,84%)	12 (63,16%)	
T3	33 (43,42%)	31 (54,39%)	0	
**pN factor**				0,02
N0	35 (46,05%)	22 (38,60%)	13 (68,42%)	
N+	41 (53,95%)	35 (61,4%)	6 (31,58%)	
**Stage**				0,01
I	9 (11,84%)	5 (8,77%)	4 (21,05%)	
IIA	26 (34,21%)	17 (29,82%)	9 (47,37%)	
IIB	15 (19,74%)	10 (17,54%)	5 (26,32%)	
III	26 (34,21%)	25 (43,86%)	1 (5,26%)	
**Induction therapy**				0,03
Yes	15 (19,74%)	8 (14,04%)	7 (36,84%)	
No	61 (80,26%)	49 (85,96%)	12 (63,16%)	
**Complications**				0,56
Pleural effusion	3 (3,94%)	2 (3,51%)	1 (5,26%)	
Vocal cord paresis	2 (2,63%)	2 (3,51%)	0	
Bronchial secretions	4 (5,26%)	3 (5,26%)	1 (5,26%)	
Wound dehiscence	2 (2,63%)	1 (1,75%)	1 (5,26%)	
Anastomotic stenosis	3 (3,94%)	1 (1,75%)	2 (10,53%)	

SCC: Squamous Cell Carcinoma; ADC: AdenoCarcinoma; VATS: Video-Assisted ThoracoScopy.

Continuous data are presented as median [range], categorical data as numbers (percentages).

Complete esophagectomy was performed in all patients: in 64 cases through a right thoracotomy, in 9 using right video-thoracoscopy, while in 3 patients (two cases of stage I and one case of stage IIA disease), at the beginning of our experience, a trans-hiatal esophagectomy was accomplished. Restoration of the alimentary tube was done in 74 cases using a narrow gastric tube; in 2 patients in whom the stomach was not considered adequate because of previous partial gastrectomy we used colon as esophageal substitute. Median length of post-operative stay was 14 days ranging between 12 and 17 days. Postoperative complications were as follow: pleural effusion in 3 patients, temporary dysfunction of the left vocal cord in 2, retention of secretions requiring fiber-bronchoscopy in 4 and thoracic wound dehiscence in 2. The cervical anastomosis was checked for leakage by roentgenograms with water-soluble contrast medium usually 5 days after surgery. Three late stenoses of the cervical anastomosis were successfully treated with endoscopic dilation. One patient developed local recurrence after two years involving the gastric tube. Eighteen patients developed distant metastases.

The overall 30-days mortality rate was of 2.6% due to 2 cases of acute mediastinitis and necrosis of the graft observed at the beginning of our experience.

Preoperative histological diagnosis was confirmed in all cases: 57 patients had squamous cell carcinoma and 19 had adenocarcinoma.

Pathologic T factor staging was as follow: 10 patients were staged as pT1, 33 as pT2 and the remaining 33 patients as pT3. A statistical significant difference was seen in pT between histopathologic types since 94% of the patients with pT3 disease had a squamous cell carcinoma (p<0.01). Forty-one patients had lymph nodes involvement at histology (pN) with a clear prevalence in the group of squamous cell carcinoma (SCC=35 versus ADC=6; *p*=0.02). The median number of lymphnodes harvested was 22 and 41 patients (53%) with involvement were treated with adjuvant chemotherapy. Radiation therapy was associated in 33 patients with T3 disease.

**Figure 1:**
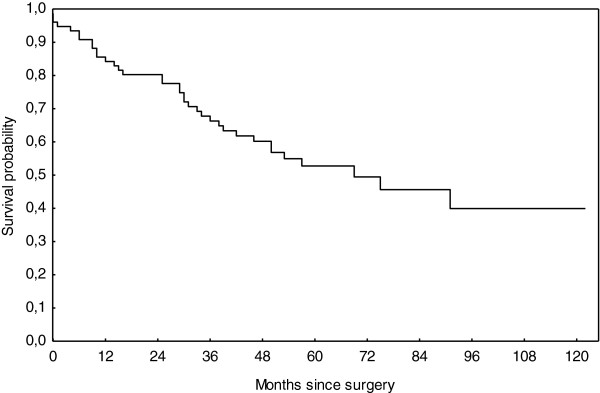
Overall survival of all patients after resection was 85.5%, 67.7% and 52.7% at 1, 3 and 5 years, respectively.

**Figure 2:**
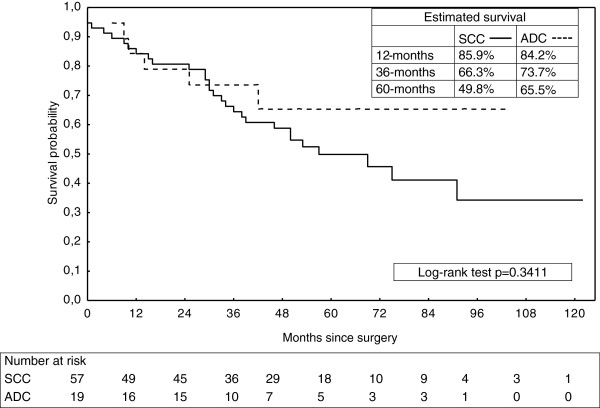
Overall survival of patients with esophageal carcinoma after resection subdivided into cell types of squamous cell carcinoma (SCC) versus Adenocarcinoma (ADC).

The median survival of the cohort was of 69 months. Overall survival was 85.5%, 67.7% and 52.7% at 1, 3 and 5 years, respectively (Figure 1). No difference in survival was found between histologic types (Figure 2).

Patients over 65 years old tended to have a worst survival after esophageal surgery for carcinomas but this difference did not reach statistical significance (*p*=0.06). Survival was better in patients with stage I or IIA tumor (*p*<0.01) independently of the histologic type. Patients that did not receive neoadiuvant treatment tended to have a better overall survival but this survival advantage reached statistical significance only in patients with stage IIB disease; *p*=0.05.

Univariate statistical analysis showed that pT1 or pT2 tumor and absence of lymph node involvement were positive predictive factors for survival after esophagectomy. The type of histology and the use of induction chemotherapy apparently did not influence survival. Not surprisingly, early stage disease (stage I or IIA) was a strong positive predictive factor for long term survival. However, on multivariate analysis, significance was found only for pT stage, N involvement, and neoadiuvant treatment while the absence of lymph node metastasis resulted as the most important predictive factor for survival (Table 2).

**Table 2:** Cox regression analysis: predictors of overall survival in patients Cohort (n=76)

**Univariate**			
	**HR**	**95% CI**	***P *value**
**T factor (T1)**	2,859	1,561 - 5,234	<0,01
**N factor (N0)**	8,606	3,262 - 22,703	<0,01
**Stage (I)**	2,503	1,688 - 3,713	<0,01
**Stage (I/IIA)**	8,983	3,423 - 23,373	<0,01
**Histology (SCC)**	0,699	0,29 - 1,684	0,427
**Neoadiuvant (No)**	1,78	0,831 - 3,815	0,139
**Multivariate**			
**T factor (T1)**	8,211	2,351 - 28,671	<0,01
**N factor (N0)**	39,076	2,605 - 586,209	<0,01
**Stage (I)**	0,277	0,059 - 1,302	0,104
**Histology (SCC)**	1,654	0,557 - 4,912	0,364
**Neoadiuvant (No)**	4,704	1,684 - 13,137	<0,01

HR: Hazard Ratio; CI: Confidence Interval.

## Discussion

This is a retrospective study conducted in a single center describing our experience in potentially curative esophagectomy and esophageal reconstruction for cancer over the last 10 years. Only few randomized trials have been published on this topic probably for several reasons: significant differences in esophageal cancer population, difficulty in standardization of the surgical technique, limited number of cases, few centers performing esophageal surgery. First of all the choice of the surgical approach is still debating.

Two major surgical strategies can be employed: en-block trans-thoracic resection or an extended resection with two or three field lymphoadenectomy. In alternative, in order to reduce the incidence of perioperative morbidity and mortality a trans-hiatal approach can be used. Several studies in fact demonstrated that the risks of respiratory complication, wound dehiscence, chylous leakage, and infection are higher after trans-thoracic esophagectomy [2-4] than trans-hiatal approach. Also the intensive care unit and the hospital length of stay are significantly longer after trans-thoracic approach.

On the other hand, [5,6] trans-thoracic esophagectomy seems associated with a significant trend toward improved long term survival. We agree with this evidence and in our series most of the patients underwent trans-thoracic esophagectomy. We performed a trans-hiatal esophagectomy only in 3 cases due to the junctional location of the tumor.

Although minimally invasive techniques are gaining in popularity, until now large randomized trials are not yet available. We performed video-assisted thoracoscopic esophagectomy only in case of early stage cancer in patients with high cardio-respiratory risk, in order to reduce the post-operative morbidity. The stomach was the method of reconstruction employed in most of the patients. We preferred to prepare a narrow gastric tube without performing piloroplasty placed in the anatomical pre-vertebral position. The advantages a narrow gastric tube are that it can be pulled up easily to the neck without tension, its excellent elasticity and reduced mediastinal encumbrance. Extra-anatomical reconstructions (retro-sternal or subcutaneous) might offer the theoretical advantage that a recurrent tumor mass will not involve the new esophagus but this choice requires a longer tube preparation. The extent of lymphnode dissection required for patients with esophageal cancer is still controversial. There are surgeons that prefer three-field lymphadenectomy, which includes abdomen, chest and neck, and proponents of the two-field lymphadenectomy, which includes abdomen and chest only. One of the potential advantages of transthoracic approach is better exposure and improved mediastinal lymphnode dissection when compared to the trans-hiatal esophagectomy. On the other hand, we preferred a two-field lymphadenectomy and notwithstanding a cervical anastomosis was performed in all cases. Although a multi-institutional trial reported that of the 30% of patients undergoing three-field lymphadenectomy with cervical lymphnode metastaases 20% occurred in patients with lower esophageal cancer, survival data are conflicting; in addiction the alleged benefit of the three-field lymphadenectomy has not been confirmed in a prospective randomized trial. Furthermore, the associated morbidity is higher ranging between 35% to 45% [7]. Another controversial issue is the site of the anastomosis in the neck or in the chest. Although proponents of intra-thoracic anastomosis point out the reduced tension and a lower risk of anastomotic complication, we prefer to use cervical anastomosis since it allows larger proximal margin of resection and in case of leakage less risks of dangerous mediastinal infection. The technique of our cervical anastomosis has been already described [8]. We prefer to accomplish a complete mechanical anastomosis. In our series we did not record any anastomotic leakage or other early anastomotic complication. Some authors reported a higher incidence of anastomotic strictures after stapled anastomosis in patients with a small diameter of the esophagus [9]. In our series, only 3 patients (4,2%) developed a late stenosis successfully treated with endoscopic dilation. Because gastric emptying may be impaired after esophagectomy and esophageal reconstruction with stomach, the use of pyloroplasty may be employed. A meta-analysis by Urschel et al. [10] showed that routine pyloroplasty is unnecessary. We agree with them and in our series we never performed pyloroplasty and we did not observe impaired gastric emptying. Furthermore, pyloroplasty might increase the risk of duodeno-gastric biliary reflux for gastric tube placed in the anatomical pre-vertebral position [11].

**Figure 3:**
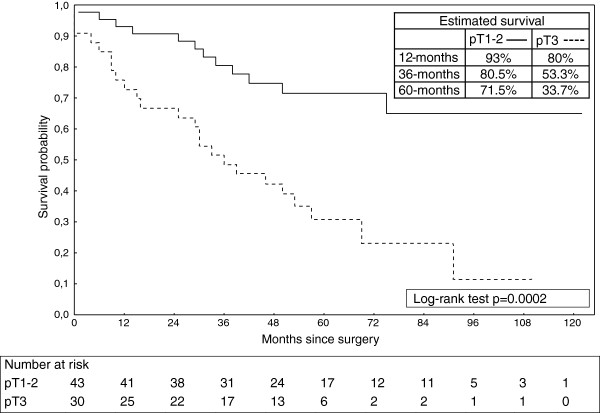
Overall survival of patients with esophageal carcinoma after resection subdivided into pT1/T2 versus pT3 tumor stage.

**Figure 4:**
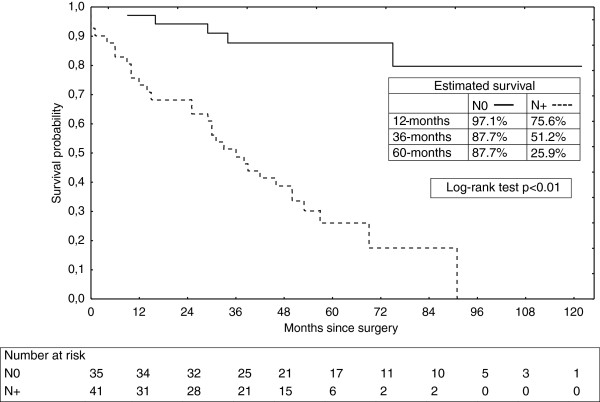
Overall survival of patients with esophageal carcinoma after resection subdivided into pN0 versus pN1 nodal stage.

At our institution, platinum based induction chemotherapy is preferred to chemo-radiotherapy in locally advanced tumors with the potential benefits of earlier treatment of micro-metastases and down-staging of tumor. Although randomized studies did not have shown a consistent benefit in term of survival [12-14] in our series 12 patients (%) presented down-staging of tumor and in 3 no residual tumor was histologically found. In this group, median survival was 38 months and 5 year survival was 44,8%. Comparison of survival between patients undergone induction chemotherapy and surgery versus surgery alone was not statistically different suggesting the efficacy of induction chemotherapy in terms of down-staging of tumor and survival. Moreover, although in the literature several studies reported an increased risk of surgical complications after induction chemotherapy [12,13], this observation was not confirmed in our series. Early pathologic tumor stage (pT) with less degree of trans-mural invasion carried a significant survival advantage (Figure 3). probably also because the prevalence of nodal metasteses increase with increasing depth of tumor penetration into the esophageal wall [15]. In our study, we have found that N0 patients had significant improvement in long term survival. (Figure 4) with a 5-year survival of 87,7%.

## Conclusions

Surgical resection is currently the treatment of choice for patients with resectable esophageal carcinoma. Although there are still several controversies regarding the optimal management of these patients, based on more than 10 year experience, our current and preferred approach is to perform a trans-thoracic esophagectomy with two-field lymphoadenctomy and cervical mechanical anastomosis. The advantages of this approach are the potential for an improved rate of complete resection, mediastinal lymphadenectomy and lower rate of anastomotic complications with a good functional outcome. In our series both T and N status appear to be the most important factors determining long term survival. Controversial issues in this field require further prospective studies to evaluate the different approaches to esophageal carcinoma.

## Competing interest

The authors declare that they have no competing interests.

## Authors’ contribution

TDG and FF conceived the study, participated in its designed and coordination and participate in surgery. PB and FV participate in surgery and helped to draft manuscript. ST and DD collected data and performed the statistical analysis. PT participated in design of the study and participated in endoscopy procedures. All authors read and approved the final manuscript.
